# Design, Implementation, Evaluation and Application of a 32-Channel Radio Frequency Signal Generator for Thermal Magnetic Resonance Based Anti-Cancer Treatment

**DOI:** 10.3390/cancers12071720

**Published:** 2020-06-28

**Authors:** Haopeng Han, Thomas Wilhelm Eigentler, Shuailin Wang, Egor Kretov, Lukas Winter, Werner Hoffmann, Eckhard Grass, Thoralf Niendorf

**Affiliations:** 1 Berlin Ultrahigh Field Facility (B.U.F.F.), Max Delbrück Center for Molecular Medicine in the Helmholtz Association (MDC), 13125 Berlin, Germany; haopeng.han@mdc-berlin.de (H.H.); thomas.eigentler@mdc-berlin.de (T.W.E.); egor.kretov@mdc-berlin.de (E.K.); 2 Humboldt-Universität zu Berlin, Institute of Computer Science, 10099 Berlin, Germany; grass@informatik.hu-berlin.de; 3 Technische Universität Berlin, Chair of Medical Engineering, 10623 Berlin, Germany; 4 Beijing Deepvision Technology Co., Ltd., Beijing 100085, China; wangshuailin@deepvision-tech.com; 5 Physikalisch-Technische Bundesanstalt (PTB), 10587 Berlin, Germany; lukas.winter@ptb.de (L.W.); werner.hoffmann@ptb.de (W.H.); 6 IHP—Leibniz-Institut für innovative Mikroelektronik, 15236 Frankfurt (Oder), Germany; 7 Experimental and Clinical Research Center (ECRC), a joint cooperation between the Charité Medical Faculty and the Max Delbrück Center for Molecular Medicine, 13125 Berlin, Germany; 8 MRI.TOOLS GmbH, 13125 Berlin, Germany

**Keywords:** thermal magnetic resonance, radio frequency heating, radio frequency signal generator, radio frequency antenna, hyperthermia

## Abstract

Thermal Magnetic Resonance (ThermalMR) leverages radio frequency (RF)-induced heating to examine the role of temperature in biological systems and disease. To advance RF heating with multi-channel RF antenna arrays and overcome the shortcomings of current RF signal sources, this work reports on a 32-channel modular signal generator (SG_PLL_). The SG_PLL_ was designed around phase-locked loop (PLL) chips and a field-programmable gate array chip. To examine the system properties, switching/settling times, accuracy of RF power level and phase shifting were characterized. Electric field manipulation was successfully demonstrated in deionized water. RF heating was conducted in a phantom setup using self-grounded bow-tie RF antennae driven by the SG_PLL_. Commercial signal generators limited to a lower number of RF channels were used for comparison. RF heating was evaluated with numerical temperature simulations and experimentally validated with MR thermometry. Numerical temperature simulations and heating experiments controlled by the SG_PLL_ revealed the same RF interference patterns. Upon RF heating similar temperature changes across the phantom were observed for the SG_PLL_ and for the commercial devices. To conclude, this work presents the first 32-channel modular signal source for RF heating. The large number of coherent RF channels, wide frequency range and accurate phase shift provided by the SG_PLL_ form a technological basis for ThermalMR controlled hyperthermia anti-cancer treatment.

## 1. Introduction

Temperature is a critical parameter of life with diverse biological implications and intense clinical interest. The aberrant thermal properties of pathological tissue have led to a strong interest in temperature as a clinical parameter. Mild regional hyperthermia (HT, T = 40–44 °C) is a potent sensitizer for chemotherapy (CH) and radiotherapy (RT), and a clinically proven adjuvant anti-cancer treatment in conjunction with RT and/or CH that significantly improves survival [1,2,3,4,5,6,7]. The clinical efficacy of hyperthermia has been demonstrated in randomized studies for specific tumor indications including RT+HT for recurrent breast cancer [8]. Vigorous fundamental and (bio)engineering research into electromagnetic radiation has resulted in a large body of literature documenting technical advances in HT devices [9]. HT devices are increasingly capable of the personalized radio frequency (RF)-induced heating of target tissue volumes guided by sophisticated treatment planning procedures and thermal dose control [10,11,12,13,14,15,16,17]. Thermal Magnetic Resonance (ThermalMR) is an HT variant that accommodates RF-induced heating [17,18,19,20,21,22,23,24,25,26], temperature mapping using MR thermometry (MRT) [26,27,28,29], anatomic and functional imaging and the option for x-nuclei MR imaging (MRI) in a single, multi-purpose RF applicator.

Targeted RF-induced heating is based on constructive and destructive interferences of electromagnetic (EM) waves transmitted with a multi-channel RF applicator. To achieve precise formation of the energy focal point, accurate thermal dose control and safety management, the transmitted RF signals’ frequency, amplitude and phase need to be regulated in real-time. Thus, the RF signal source is the key component for facilitating appropriate frequency, amplitude and phase settings of the RF signals. The radiation pattern of the single RF transmit element, the RF channel count and the RF frequency of the RF applicator are of high relevance for ensuring a patient and problem-oriented adaptation of the size, uniformity and location of the RF energy deposition in the target region [19,21,22,24,30]. The (re)design of multi-channel RF applicator configurations showed more than twofold enhancement of the RF power focusing capability by increasing the number of RF antennae from 12 to 20 [31,32]. Increasing the number of RF antennae resulted in higher RF power absorption and enhanced tumor coverage ratios in deep-seated brain tumors in children [33]. The optimal operating RF frequency depends on the RF applicator characteristics and the target tissue parameters [34]. Lower RF frequencies focus EM energy to larger regions and have lower energy losses inside and outside tissue. Higher RF frequencies facilitate focusing EM energy onto small targets. Numerical simulations and evaluation studies investigated the optimal RF frequency [24]. For regional hyperthermia improvement of the RF power absorption in the target region versus regions outside the target was demonstrated when increasing the RF frequency from 100 MHz to 150 MHz and 200 MHz [35,36]. The optimal heating frequency was examined for seven tumor locations using RF frequencies ranging from 400–900 MHz [37]. For superficial tumors, the highest average specific absorption ratio (aSAR) was obtained with higher frequencies where aSAR was improved with increasing the number of RF antennae. For deep-seated tumors, the highest aSAR was reported for lower frequencies. Studies on ultimate SAR amplification factors and RF applicator concepts suggested the use of high frequencies up to 1 GHz for a highly focused EM energy deposition [21,30]. Time-multiplexed beamforming, a mixed frequency approach and multi-frequency SAR focusing provide other directions into optimization of RF heating performance [38,39,40]. Recently, an iterative multiplexed vector field shaping (MVFS) approach was introduced to solve the time- and frequency multiplexed problem of constrained RF-induced hyperthermia [24]. This work underlined the need of wideband signal generators by demonstrating the contribution of distinct frequencies to the RF heating and by showing that these frequencies and contributions depend on the target geometry.

To summarize, advancing high-fidelity RF hyperthermia requires pioneering strategies that exploit a wider range of RF frequencies and high-density RF antenna arrays with miniaturized RF building blocks that permit independent frequency, amplitude and phase control for each channel. Hardware implementations for RF heating typically operate at a fixed frequency and have a limited number of RF channels [10,32,41,42,43,44,45,46,47]. Recognizing these opportunities and challenges, this work reports on the design, implementation, evaluation, validation and application of a 32-channel modular signal generator (SG_PLL_) that uses phase-locked loop (PLL) circuit blocks and permits high amplitude, phase and frequency tuning resolution. This setup is designed to operate between 0.06–3.0 GHz and can be used as the signal source for ThermalMR. To our knowledge, this is the first PLL based multi-channel modular signal source for RF heating with frequency, phase and amplitude adjustment functions integrated.

## 2. Results

### 2.1. System Characterization

**Figure 1:**
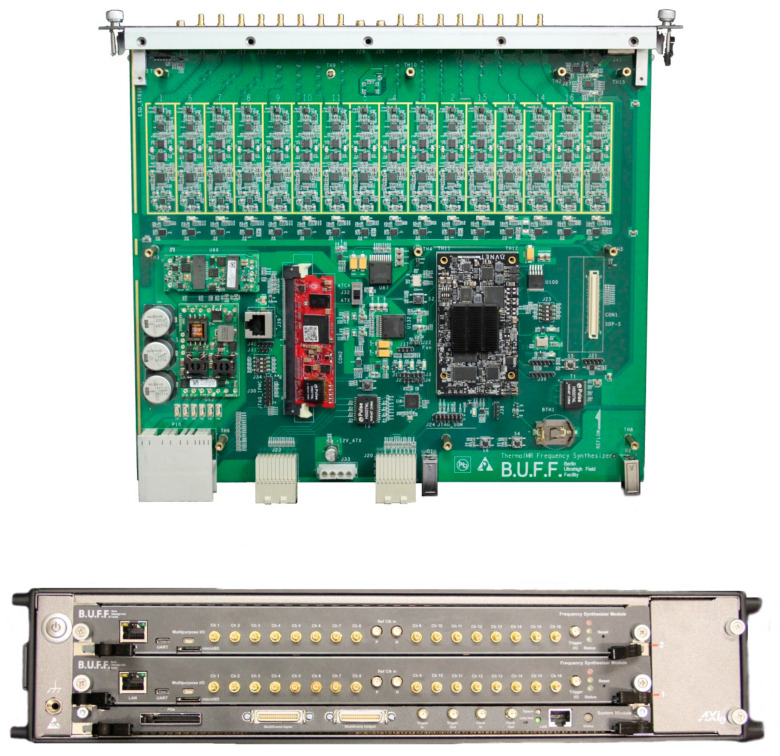
Sixteen-channel frequency synthesizer module (top): The black module is a system-on-module unit AES-ZU3EG-1-SOM-I-G (Avnet, Phoenix, AZ, USA); the red module is an open source intelligent platform management controller card. Electromagnetic interference shielding covering the 16 RF channels was not installed for acquisition of the photo. The top cover of this module was also removed for better presentation. Two 16-channel frequency synthesizer modules installed in the AXIe chassis are shown at the bottom.

Implementation of the designed 32-channel RF signal generator was successfully performed with the two 16-channel frequency synthesizer modules integrated in the AXIe (Advanced Telecommunications Computing Architecture (ATCA) Extensions for Instrumentation and Test [48]) chassis as illustrated in Figure 1. The hardware module has a size of (284 × 322 × 29) mm^3^ and a weight of 2.58 kg including the front panel and the protection covers. The red intelligent platform management controller (IPMC) mezzanine card [49] was included to comply with the AXIe-1 specification [48]. It communicates with the AXIe system module in the chassis for tasks such as power management, temperature monitoring, backplane pin assignment, etc. A set of fans is integrated in the chassis to dissipate heat generated by the modules. The power consumption of one module was measured to be 7.32 W with a CPU usage of 100% and the RF section switched off. Typical power consumption was 50.42 W when all the 16 RF channels were outputting 0 dBm signals. When the power levels of all the 16 RF channels were set to −4 dBm, the total power consumption started to exceed 50 W which is the maximum allowed power consumption for one unmanaged AXIe module [48]. Peripheral circuits working with the IPMC card were implemented for temperature probing of the module and for adjustment of the chassis (fan speed, etc.) accordingly in case the power consumption for one module exceeds 50 W. 

**Figure 2:**
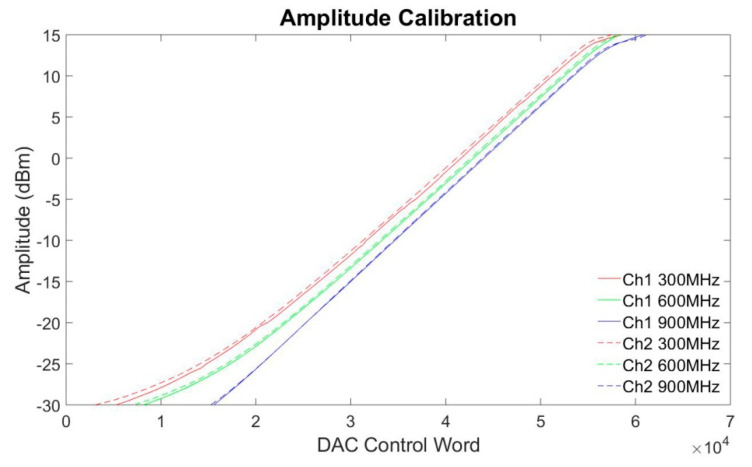
Amplitude calibrations for two channels at three frequencies: 300 MHz, 600 MHz and 900 MHz.

Videos S1–S3 demonstrate live adjustments of the signal frequency, amplitude and phase. Videos S4–S6 show the switching/settling time measurements of frequency, amplitude and phase with the results detailed in Table 1. Amplitude calibrations were conducted for channel one and channel two for three frequencies: 300 MHz, 600 MHz and 900 MHz. Figure 2 shows the relationship between the digital to analog converter (DAC) control words and the signal amplitudes. Good linearity was achieved for power levels between −25 dBm to 10 dBm. It requires a larger control word to output the same power level for a higher frequency. This complies with the characteristics of the voltage controlled variable gain amplifier (VGA) chip ADL5330 [50]. Table 2 lists the test results of the phase shift experiments. An average absolute phase shift error of 0.06° with a maximum phase shift error of 0.16° was measured for all tested cases. The phase shift error showed no dependency on the phase shift value. Test results at 900 MHz showed slightly higher phase errors versus phase errors obtained at lower frequencies. 

### 2.2. E-Field Manipulation and Mapping

**Figure 3:**
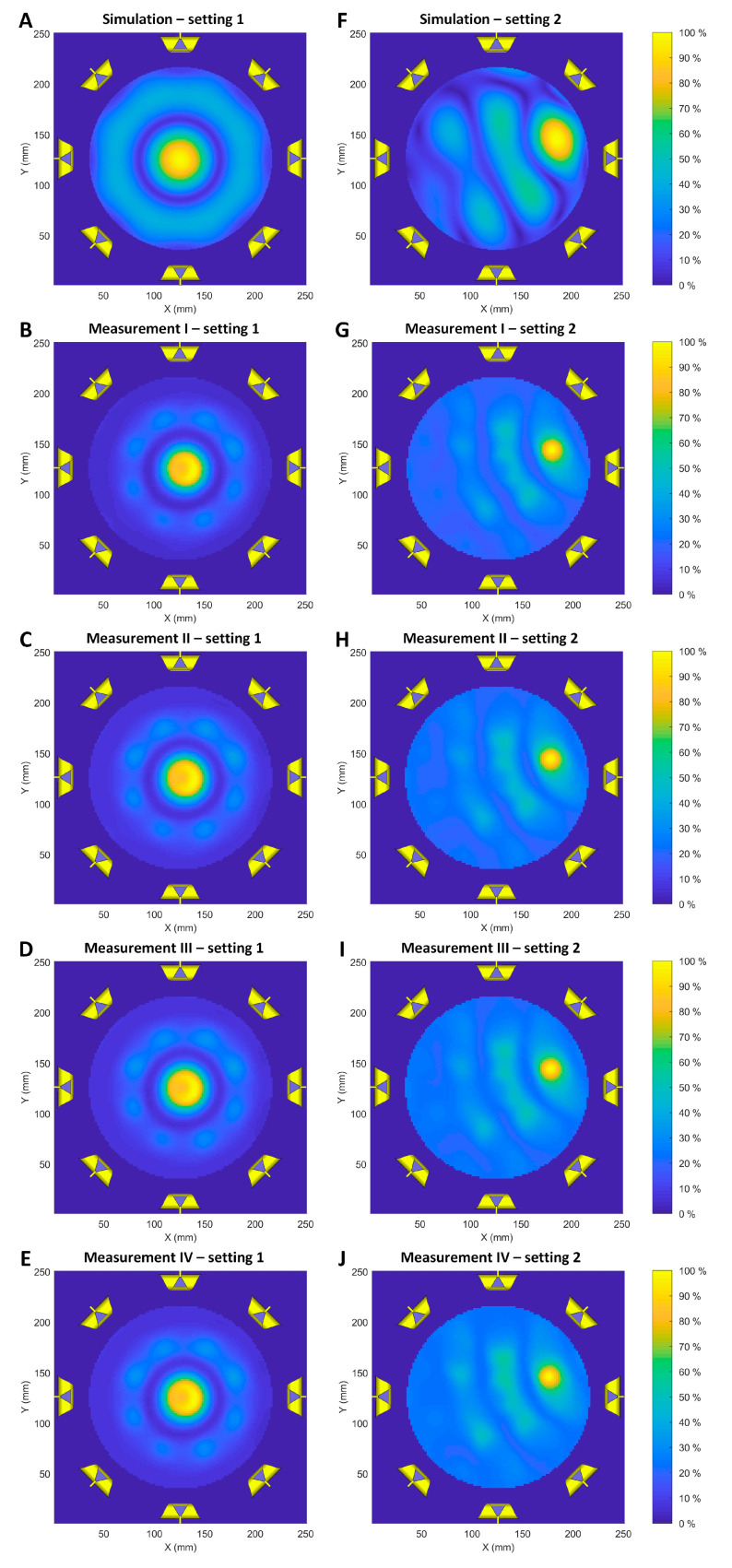
E-field simulations and measurements (f = 400 MHz) obtained for the central plane of the self-grounded bow-tie (SGBT) antennae array. (**A**–**E**) Normalized E-field maps with the E-field focus being placed in the center of the transversal plane through the middle of the SGBT antenna array. (**F**–**J**) Normalized E-field maps with the E-field focus being positioned off-center in the same transversal plane used for the center position. Two phase and amplitude settings were tested in the simulations and measurements. All eight RF channels were set to the same phase (0°) and amplitude (10 dBm) in setting 1. In setting 2, the phases of the eight RF channels were set to [6.04°, −154.96°, 25.86°, −32.9°, −178.5°, −7.46°, −3°, −155.89°] and the amplitudes were set to [−1.15 dBm, −14.11 dBm, −13.01 dBm, −3.72 dBm, 2.22 dBm, 10 dBm, 9.2 dBm, 3.32 dBm]. A different set of eight out of 32 RF channels was used for measurement I–IV.

Electric field (E-field) simulations and measurements (f = 400 MHz) are shown in Figure 3 for two E-field focusing point settings. For this purpose, the E-field maps obtained from simulations and measurements were normalized. For all eight RF channels using the same RF phase and amplitude setting, the E-field focusing point is located at the center of the transversal plane through the middle of the antenna array (Figure 3A–E). Figure 3F–J show the results obtained for positioning the E-field focus in an arbitrary (off-center) location. The measured E-field distribution patterns agree with the E-field maps derived from the electromagnetic field (EMF) simulations. The same E-field distribution was observed in all four experiments, each using a different set of eight out of 32 RF channels. 

### 2.3. Single Channel RF Heating

**Figure 4:**
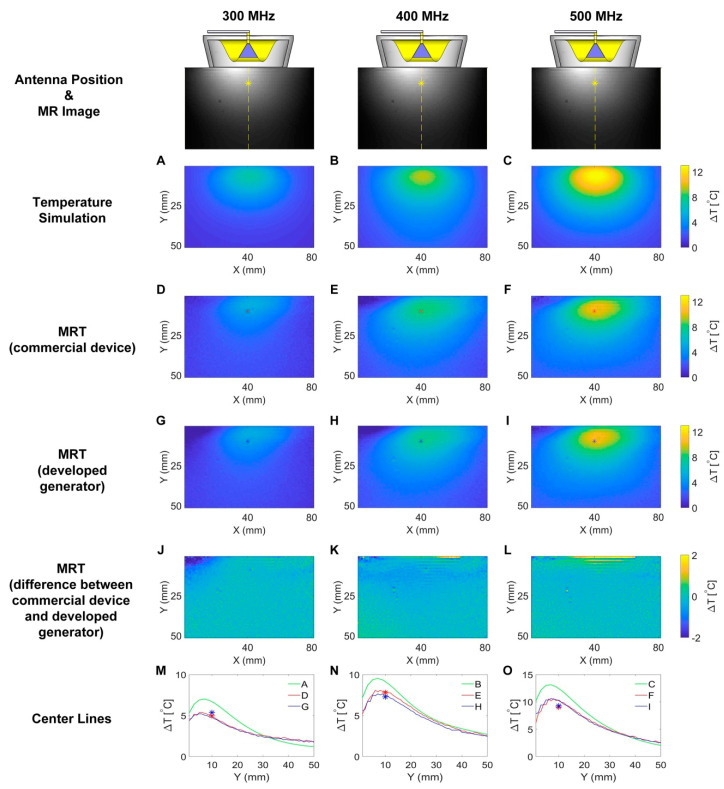
Maps of temperature changes (ΔT) obtained from the numerical simulations and experiments using a single channel connected to an SGBT antenna for RF heating (t = 10 min, P_in_ at port = 17.78 W). Each column shows results for one frequency: 300 MHz, 400 MHz and 500 MHz. A transversal slice in the middle of the phantom aligned with the center of the SGBT antenna was selected for the temperature simulations and for MR thermometry (MRT). The first row demonstrates the position of the antenna and the MR image of the phantom. The yellow lines indicate the center lines and the yellow stars indicate the position of the fiber optic temperature sensor. Figure (**A**–**C**) illustrate temperature changes obtained from temperature simulations. Figure (**D**–**F**) show maps of temperature changes derived from MRT for RF heating using the commercial signal generator (SMGL, R&S, Munich, Germany). The red stars in these figures indicate the position of the fiber optic temperature sensor. Figure (**G**–**I**) depict maps of temperature changes deduced from MRT of RF heating using the signal generator developed in this work. The blue stars in these figures represent the position of the fiber optic temperature sensor. Figure (**J**–**L**) outline ΔT difference maps benchmarking the temperature changes obtained for RF heating using the proposed signal generator against those observed for the commercial SMGL signal generator. The bottom row (Figure (**M**–**O**)) show ΔT profiles obtained for a center line placed across the center slice of the phantom for temperature simulations (**A**–**C**), experimental RF heating using the commercial SMGL signal generator (**D**–**F**) and experimental RF heating employing the RF signal generator setup developed in this work (**G**–**I**). The blue stars and red stars indicate readings from the temperature sensor for heating with our signal generator and with SMGL, respectively.

The results deduced from single channel RF heating in numerical temperature simulations and experiments are detailed in Figure 4 for f_1_ = 300 MHz, f_2_ = 400 MHz and f_3_ = 500 MHz. RF at higher frequencies induced higher temperature changes (ΔT) in the phantom. Figure 4M–O depict ΔT profiles obtained for a center line placed across the transversal slice at the middle of the phantom for temperature simulations (Figure 4A–C), experimental RF heating using the commercial SMGL (R&S, Munich, Germany) signal generator (Figure 4D–F) and experimental RF heating employing the RF signal generator developed in this work (Figure 4G–I). Figure 4J–L show the differences in RF-induced temperature changes obtained from MRT for the developed signal generator (Figure 4G–I) and the commercial SMGL signal generator (Figure 4D–F). Almost identical temperature changes across the phantom were observed. The maximum temperature increase derived from MRT was ΔT = 5.3 °C, 7.8 °C and 10.6 °C for heating at 300 MHz, 400 MHz and 500 MHz. These temperature changes are 1.7 °C, 1.7 °C and 2.5 °C lower than the corresponding maximum temperature increases yielded by the numerical temperature simulations. Fiber optic temperature measurements confirmed the MRT results.

### 2.4. Dual Channel RF Heating

**Figure 5:**
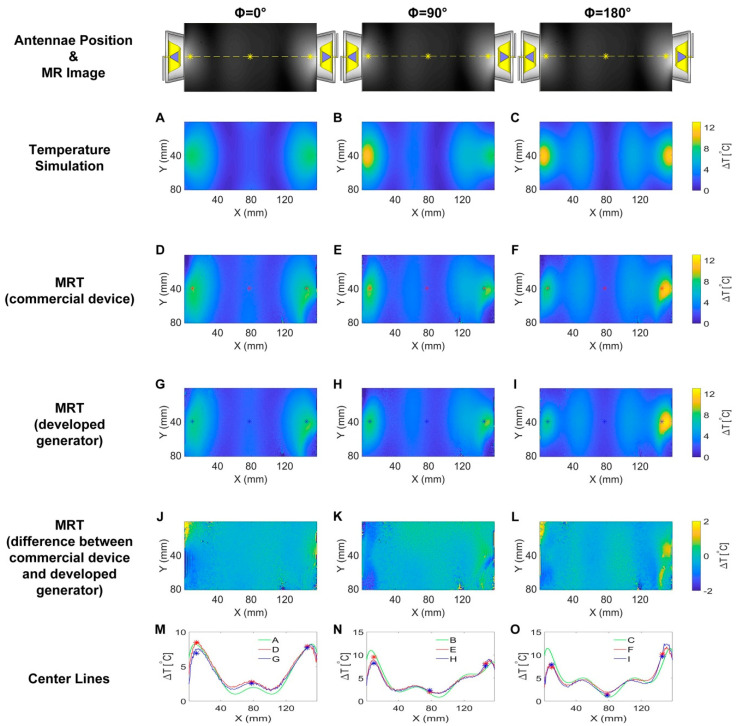
Maps of temperature changes (ΔT) obtained from the numerical simulations and experiments using two channels (f = 400 MHz) for RF heating (t = 10 min, P_in_ at port = 17.78 W) with each channel being connected to an SGBT antenna. The first row demonstrates the position of the antennae and the MR image of the phantom. The yellow lines indicate the center lines and the yellow stars indicate the position of the fiber optic temperature sensor. Each column shows results obtained for one phase setting: ϕ = 0°, ϕ = 90° and ϕ = 180°. The phases were set to the right channel while the left channel was fixed to ϕ = 0°. A transversal slice in the middle of the phantom aligned with the center of the RF applicator was selected for MR thermometry. Figure (**A**–**C**) illustrate temperature changes obtained from temperature simulations. Figure (**D**–**F**) show maps of temperature changes derived from MRT for RF heating using the commercial signal generator (M8190A, Keysight). The red stars in these figures indicate the position of the fiber optic temperature sensor. Figure (**G**–**I**) depict maps of temperature changes deduced from MRT of RF heating using the signal generator developed in this work. The blue stars in these figures represent the position of the fiber optic temperature sensor. Figure (**J**–**L**) outline ΔT difference maps benchmarking the temperature changes obtained for RF heating using the proposed signal generator against those observed for the commercial M8190A signal generator. The bottom row (Figure (**M**–**O**)) show ΔT profiles obtained for a center line placed across the center slice of the phantom for temperature simulations (**A**–**C**), experimental RF heating using the commercial M8190A signal generator (**D**–**F**) and experimental RF heating employing the RF signal generator setup developed in this work (**G**–**I**). The blue stars and red stars indicate readings from the temperature sensor for heating with our signal generator and with M8190A, respectively. Constructive interference patterns were observed in the middle of the phantom for phase setting ϕ = 0°. This pattern was shifted ~18 mm to the left for phase setting ϕ = 90°. For phase setting ϕ = 180°, two symmetrical constructive interference patterns ~64 mm apart from each other and a destructive interference pattern in the middle of the phantom were observed.

Figure 5 summarizes the results derived from numerical simulations and experiments using the two-channel RF heating setup at 400 MHz. Figure 5J–L depict the difference in the temperature changes obtained with MRT for RF heating using proposed signal generator (Figure 5G–I) and the commercial M8190A (Keysight, Santa Rosa, CA, USA) arbitrary waveform generator (Figure 5D–F). Similar to the single channel experiments, almost identical temperature changes across the phantom were observed. Temperature profiles obtained from center lines across the phantom (Figure 5M–O) demonstrated the same interference patterns created in the experiments compared with the simulations. For phase setting ϕ = 0°, the induced temperature increase due to constructive interference was ΔT_max_ = 2.6 °C in the middle of the phantom (Figure 5A,D,G). This interference pattern was moved around 18 mm to the left towards channel two when a 90° phase shift was applied to channel one on the right (Figure 5B,E,H). A destructive interference was created in the middle of the phantom when the phase difference was set to 180° between the two channels. For this phase mode two constructive interferences around 64 mm apart from each other were generated symmetrically in the phantom (Figure 5C,F,I). Readings from the fiber optic temperature sensor accord with the MRT results.

## 3. Discussion

### 3.1. System Characterization

The 32-channel signal generator consists of two PLL-based modular frequency synthesizers. This modularity and the working principle of PLLs support convenient implementation of *n* > 32 number of RF channels. In a ThermalMR setting, the RF signal could potentially come from the MR scanner; however, the current maximum number of independent transmission RF channels in a state-of-the-art MR scanner is constrained to a single TX channel for the combined mode transmission regime and eight or sixteen for the parallel transmission mode with RF signals being constrained to a small transmitter bandwidth covering a fixed center frequency (Larmor frequency). It is fair to anticipate that the number of transmitter RF channels will increase to meet the needs of ThermalMR which would be in favor of small size antenna building blocks at higher frequencies, which afford high density RF applicator configurations. Previous experimental works mostly operated at one of the ISM (Industrial, Scientific and Medical) frequencies (e.g., 434 MHz, 915 MHz and 2.4 GHz) and typically a single channel commercial signal source in conjunction with an RF power splitter and RF phase shifters architecture was adopted [43,47]. ThermalMR exploits a wider frequency range with the proposed signal generator which covers a wide frequency range from 60 MHz to 3 GHz. The compact modular PLL based design implemented in this study provides a theoretically unlimited number of coherent, independent RF channels and a wide frequency range, thus facilitating future ThermalMR developments.

Live adjustments of the RF signal were demonstrated in Videos S1–S3. The high-performance field-programmable gate array (FPGA) chip makes it possible to carry out adjustments through executing Python scripts on its ARM processor. Unstable signals were observed in Videos S4–S6 during transitions. These unstable transitions can be bypassed by setting the RF switch chips included in the signal path. No significant delay is added by this approach since the switching time of the RF switch chips is typically as low as 150 ns [51]. The switching/settling times are sufficient for ThermalMR applications. Especially, the fast and stable signal switching/settling implemented here is necessary for ThermalMR applications where real-time signal adjustments are needed, e.g., in online interference pattern control [52,53], in time-multiplexed beamforming [38], or in a mixed frequency approach [39]. Fast switching is also desirable during system initialization where recursive adjustments could be involved. However, generating excitation RF pulses suitable for MRI typically requires an amplitude settling time of less than 0.1 μs, which is a recognized limitation of the developed 32-channel RF signal generator. 

In Figure 2, a slight difference between channels on the control words for generating the same signals was observed. This difference was produced by the variations of the components on the RF signal path. A larger control word is required to output the same power level for a higher frequency. The dependency of the control words on system parameters such as frequency and RF channel can be eliminated by applying a power meter and a feedback control algorithm. A home-built multi-channel power and phase meter was developed to implement more precise control over the output signal amplitude.

In the phase shift experiments, test results (Table 2) at 900 MHz showed slightly higher phase errors versus phase errors obtained at lower frequencies. This is due to the higher phase jitter of the PLL’s output at higher frequencies. The starting phases after synchronization before phase shifting were different for the tested frequencies. It was mainly caused by the difference in the signal path lengths between the two channels. This phase mismatch can be synchronized/compensated by applying the phase meter and a synchronization algorithm. The high accuracy of the phase shifting function of the signal generator is essential for ThermalMR applications since the desired focal point formation highly depends on precise phase arrangement of transmitted RF signals. This PLL-based phase shifting approach showed substantial improvement in flexibility, resolution, feasibility and accuracy over other approaches such as standalone phase shifter based and modulation-based phase shifting. For example, an 8-bit digital phase shifter (DST-10-480/1S, Pulsar Microwave, Clifton, NJ, USA) was used in an experimental setup [44]. It can only generate 256 phases and covers a limited frequency range. For this approach, the maximum insertion loss is as high as 6 dB and the phase uncertainty is ±3% at center frequency [54]. For modulation-based phase shifting [55], the resolution and the accuracy of the phase shift are usually confined by the number of points in the modulation constellation diagram and the accuracy of the DACs. DDS based signal generator could provide accurate phase shift with fine resolution. However, the maximum output frequency of a DDS is limited to about 1/3 the sampling clock frequency [56]. If higher frequencies are needed, harmonics in higher Nyquist zones need to be filtered out with band-pass filters. It is difficult to cover a wide frequency range with a DDS system. Here, we addressed these constraints by employing the PLL based signal synthesizing approach which provides a wide frequency range (0.06–3 GHz) and fine phase adjustment resolution (360°/2^24^).

### 3.2. E-Field Manipulation and Mapping

The controlled deposition of electromagnetic energy in the target is essential to RF-induced hyperthermia. The results obtained from the E-field manipulation experiments demonstrate the signal generator’s capability of controlling the RF signals to generate desired E-field patterns based on constructive and destructive interferences of electromagnetic waves. Our approach demonstrated that all 32 RF channels were functioning correctly in synthesizing the desired E-field patterns. The E-field amplitudes derived from the measurements are inferior to the E-field simulations. This difference is caused by the nonlinear sensitivity of our home-built E-field probe. 

### 3.3. Single Channel RF Heating

Maps of temperature changes (ΔT) obtained from the numerical simulations and experiments using a single channel connected to a self-grounded bow-tie (SGBT) antenna for RF heating showed that RF at higher frequencies induced higher ΔT in the phantom. This is caused by the higher loss of the RF signal in the phantom at higher frequencies. The MRT results revealed ΔT profiles which are in accordance with the simulation results. The maximum temperature increases derived from MRT were lower than the corresponding maximum temperature increases yielded by the numerical simulations. This difference is caused by the changeover time (Δt = 50 s) needed to switch the cable connection from the home-built RF power amplifier (RFPA) to the MR scanner right after the heating process. The temperature in the phantom drops due to heat dissipation during this change over time.

### 3.4. Dual Channel RF Heating

A second channel was added to the single channel RF heating experiments to examine interference patterns created by phase shifts. Similar to the single channel experiments, almost identical temperature changes across the phantom between the designed signal generator and the high-end commercial one were observed. Temperature profiles obtained from center lines across the phantom underline the equivalence in RF heating performance of the proposed signal generator and the M8190A. The interference patterns created by RF heating are in accordance with numerical temperature simulations. The differences between MRT and temperature simulation have a slightly different pattern compared with corresponding differences obtained for the single channel heating experiments. This was caused by the imperfection of the RFPAs whose outputs were impacted by the crosstalk (which depends on the phase settings) between the two SGBT antennae.

To summarize, the experimental results demonstrated the suitability of the designed signal generator for ThermalMR. Compared with commercial signal generators, this design provides large number of channels and various communication interfaces implemented here make it very convenient to be integrated into a more complex system, e.g., an MR scanner. The high-performance processor adopted in the design provides ample processing power for applications that need real-time signal adjustments and flexible configurations. The PLL circuit implemented in this design occupies little printed circuit board (PCB) area and permits a compact modular design. The adoption of PLL is also cost-effective compared to other architectures of comparable performance. Although the maximum frequency tested here was 1.2 GHz, the signal generator supports higher frequencies up to 3 GHz. This wide frequency range extends its usage from RF-induced mild hyperthermia to microwave ablation [57]. 

## 4. Materials and Methods 

### 4.1. Hardware Design

**Figure 6:**
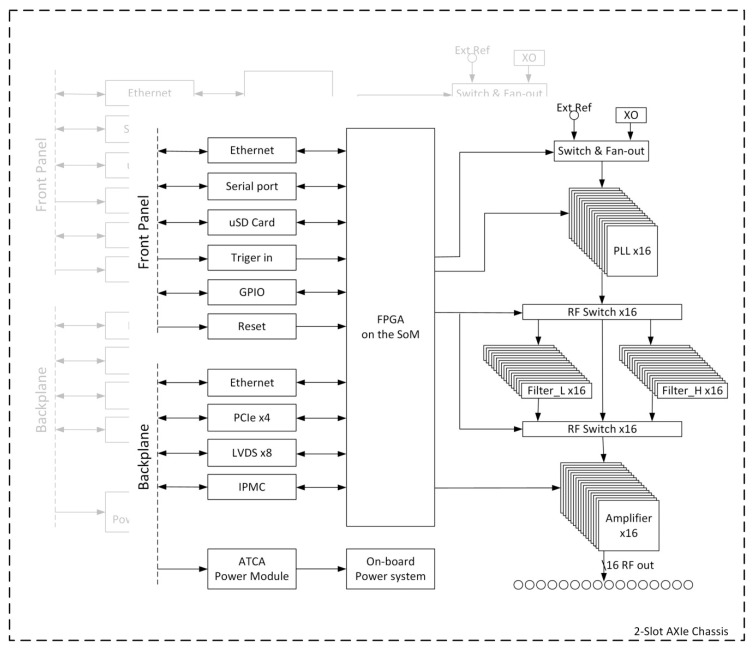
System block diagram of the 32-channel RF signal generator. Two 16-channel AXIe RF signal synthesizer modules were installed into a 2-slot AXIe chassis to form a 32-channel RF signal generator. The modules communicate with each other through the backplane LVDS connections. The chassis provides power to the modules through its backplane.

The 32-channel RF signal generator hardware consists of two 16-channel RF synthesizer modules. Figure 6 shows its block diagram. The 16-channel RF synthesizer module is an AXIe compliant modular design. The 16 RF channels are identical in circuit design with the output impedance matched to 50 Ohm. Each channel is equipped with an independent low-dropout power regulator that powers the noise sensitive components in the channel. The RF signal generation was designed around the phase-locked loop chip ADF4356 (Analog Devices, Norwood, MA, USA). This PLL chip could generate a frequency range of 54 MHz to 6800 MHz. A very fine frequency resolution with practically no residual frequency error is afforded by the PLL’s 52-bit modulus. The synthesized signal’s phase can be adjusted with a theoretical resolution of 360°/2^24^. Two low-pass filters with a bandwidth of 400 MHz and 1.2 GHz were added to filter out the harmonics of the RF signals. RF switch chips HMC245A (0 to 3.5 GHz, Analog Devices) were used to select among different filter paths. The signal amplitude can be manipulated by a voltage controlled variable gain amplifier (VGA) chip ADL5330 (10 MHz to 3 GHz, Analog Devices) which provides a wide gain control range. The gain of the VGA is adjustable linearly in decibel and was controlled by the voltage output of a 16-bit digital to analog converter (DAC) AD5683 (Analog Devices). The 16 PLL chips on the module were locked to the same reference signal. A low jitter 2-input selectable 1:16 clock buffer CDCLVP1216 (Texas Instruments, Dallas, TX, USA) was used to fan out the reference signal to 16 PLL chips. The reference signal can be selected either from the output of an on board programmable low jitter crystal oscillator Si549 (Silicon Labs, Austin, TX, USA) or from the external reference signal input. The routings of the LVPECL (low-voltage positive emitter-coupled logic) reference signals for the PLLs as well as the routings of the signals from the output of the VGA to the SMB (subminiature version B) connectors at the board edge were length matched with minimum variations among the 16 channels.

The whole system was managed by a quad-core ARM Cortex-A53 processor which resides in a field-programmable gate array chip ZU3EG (Xilinx, San Jose, CA, USA). The FPGA seats at the core of a system-on-module unit AES-ZU3EG-1-SOM-I-G (Avnet, Phoenix, AZ, USA). Various interfaces were implemented with the FPGA: a Gbit Ethernet port, a serial port, an SD (secure digital) card interface, GPIO (general purpose input/output) connections, three status LED (light-emitting diode) indicators, trigger input/output and a reset input were connected to the front panel; a Gbit Ethernet port, a 4-lane PCIe (peripheral component interconnect express) port and 4-lane LVDS (low-voltage differential signaling) signals were connected to the backplane per the requirements of the AXIe specification [48]. The Ethernet interface to the front panel, the PCIe port, the SD card interface and the serial port were implemented with the hard-core peripherals within the processor system whereas the rest of the interfaces were realized using the programmable logic resources in the FPGA. AXI (Advanced Extensible Interface) bus-based IP (intellectual property) cores were developed utilizing the FPGA logic to configure the PLL chips, RF switches, DAC chips and the clock buffer.

This 16-channel RF synthesizer module works with any AXIe compatible chassis. Commercial-off-the-shelf chassis were used to save the effort of designing the data exchange mechanism, communication interfaces, power supply and cooling system. Two modules were installed into a 2-slot AXIe chassis M9502A (Keysight, Santa Rosa, CA, USA) to form a 32-channel RF signal generator. The modules communicate with each other through the backplane LVDS connections. The module in the lower slot is a master module and controls the other one. An open source intelligent platform management controller (IPMC) mezzanine card [49] can be installed into the mini dual in-line memory module (DIMM) socket on the AXIe module. The chassis provides power to the modules through its backplane. An ATCA power input module PIM400KZ (ABB, Zurich, Switzerland) was used to interface the −48 V DC (direct current) power supply from the backplane. A DC-DC converter ESTW010A0B (ABB) converts the −48 V DC to 12 V DC which then served as the main power supply of the module. A 12 V DC power input socket was also implemented on the module to enable standalone operation.

### 4.2. Software Design

**Figure 7:**
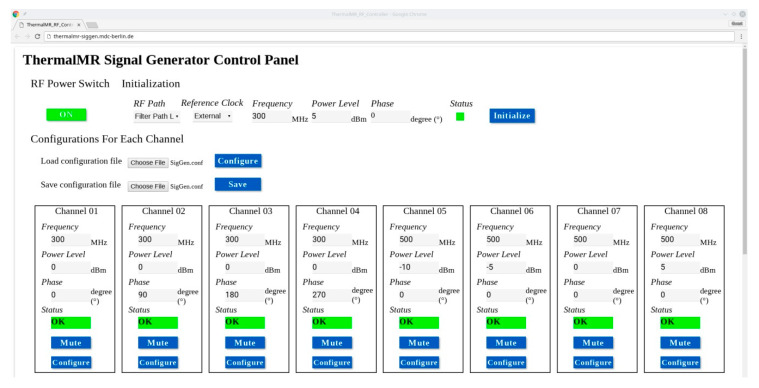
The web-based graphic user interface used for controlling the 32-channel signal generator. For simplicity only eight out of 32 channels are shown. The RF Power Switch controls the power supply to the analog circuits in the signal generator. The status indicator shows green when the corresponding channel runs normally. If errors happen, the indicator turns into red. Each channel can be muted independently by pressing the Mute button.

The module is running openSUSE LEAP 15.1 Linux operating system [58]. Python scripts were programmed to interact with the IP cores which control the RF components on the board. The programs manipulate the IP cores through memory mapped register reading and writing. A web based graphical user interface was developed to provide a more user-friendly interface to configure the system as demonstrated in Figure 7. All the 32 RF channels can be set to the same configuration specified by the user in the initialization function block. System-wide options, e.g., reference clock selection and filter path selection were also implemented in this block. Parameters can be loaded from/saved to configuration files so that the configuration process is less tedious and errors from manual input can be avoided. Each channel can be configured individually in the following blocks. Signal properties such as frequency, phase and amplitude are set according to the parameters specified in the input text boxes.

### 4.3. System Characterization 

For system evaluation, the reference clock for the PLLs was provided by a GPS (global positioning system) disciplined oscillator’s output distributed by an 8-channel clock distributor CDA-2990 (National Instruments, Austin, TX, USA). The power level of the PLL output was set to 5 dBm. All tests were conducted at room temperature (22 °C) with the signal generator warmed up for 30 min.

The power consumption of the module was measured in standalone operation mode with all the RF components being powered on and off. FPGA logics and Python scripts were implemented to test the module’s ability of manipulating the frequency, amplitude and phase of the RF signals. Maximum switching/settling times for changes in these properties were examined. A single pulse generated by a universal pulse generator (UPG100, ELV, Leer, Germany) was used to trigger the change. The trigger signal (via a T-connector) and the RF output from the module were connected to an oscilloscope (DPO7254, Tektronix, Beaverton, OR, USA) to measure the switching/settling times. In total, 50 measurements with 10 frequency points ranging from 100 MHz to 1000 MHz in increments of 100 MHz were conducted for testing the PLL frequency lock time. Amplitude changes (*n* = 18 measurements) were assessed for a range of −30 dBm to 15 dBm with a step size of 5 dBm for testing the amplitude switching/settling time. Various phase changes were also carried out for testing the phase switching time.

The signal amplitude of each channel is controlled by the combination of a DAC and a VGA. Amplitude calibration was conducted to map 16-bit DAC control words to specific signal power levels. Signals at three frequencies (300 MHz, 600 MHz and 900 MHz) were calibrated. The RF power level was monitored with a spectrum analyzer (ZVL, R&S, Munich, Germany). A Python script was developed to change the 16-bit control word of the DAC. The control words were recorded for 91 power levels spread over −30 dBm to 15 dBm with a step size of 0.5 dBm.

The accuracy of the phase shifting of the PLLs was tested employing the phase measurement function of an oscilloscope (MSOS054A, Keysight). The RF signal generated from channel one of the module was used as a reference signal. Various phase shift settings were applied to channel two. The phase relationships between the two channels were recorded before and after the phase shifting. Signals at three frequencies (300 MHz, 600 MHz and 900 MHz) were tested.

### 4.4. E-Field Manipulation and Mapping

**Figure 8:**
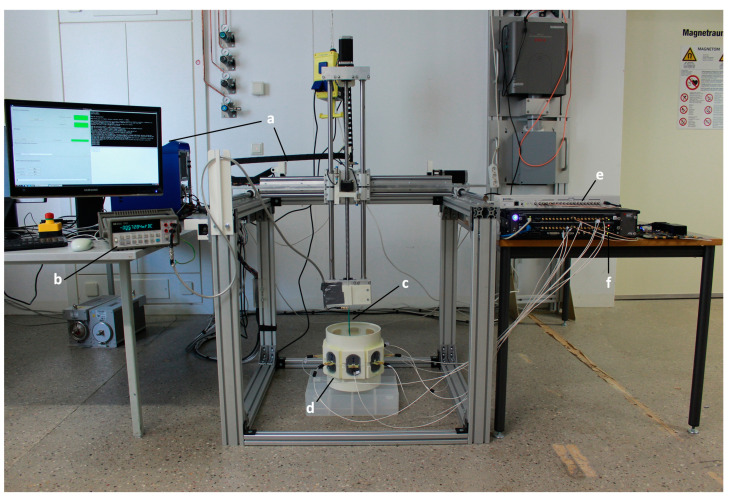
Experimental setup used for E-field mapping. (**a**) the COSI Measure setup, (**b**) digital multimeter (34401A, Keysight) connected to (**c**) the home-built E-field probe, (**d**) 8 SGBT antennae arranged in a circular array (diameter = 22 cm) immersed in deionized water, (**e**) clock distributor, (**f**) RF signal generator. COSI Measure moves the E-field probe with a step size of 2 mm in the central transversal plane of the antennae setup resulting in 6430 measurement points.

E-field manipulation and E-field mapping were conducted in EMF simulations and in experiments to demonstrate the signal generator’s performance for E-field focusing. For this purpose, eight arbitrary RF channels (four from each module of the signal generator) were used to generate 400 MHz RF signals using tailored amplitude and phase settings. The RF signals were connected to eight wideband self-grounded bow-tie (SGBT) antennae [23] immersed in deionized water. The SGBT antennae were arranged in a circular array. An open source 3D multipurpose measurement system (COSI Measure) [59] and a home-built E-field probe were used to map the E-field distribution. Figure 8 illustrates the experimental setup. Two E-field patterns: (a) the E-field focusing point was placed in the center of the transversal plane through the middle of the SGBT antenna array; (b) the E-field focusing point was set to an arbitrary point in the transversal plane through the middle of the SGBT antenna array. The amplitude and phase settings for each E-field pattern were obtained from an alternating projections-based EM field optimizer [60]. The amplitude and phase settings were fed to the signal generator for E-field mapping and to CST Microwave Studio 2018 (Computer Simulation Technology GmbH, Darmstadt, Germany) for E-field numerical simulation. For the simulation, the CST frequency domain solver was adopted with a tetrahedral mesh type. The maximum mesh size was set to 4.0 × 4.0 × 4.0 mm³ including an adaptive mesh refinement to improve the mesh quality. The mesh size is sufficient for the problem since further reduction (10%) of the maximum mesh size did not yield substantial changes (<0.3%) in the simulation results. We used open boundary condition which is implemented as a perfectly matched layer (PML) with additional 3 m (4 wavelengths at 400 MHz) distance added between the model and the PML. The experiment was repeated four times. For each run, a different set of eight RF channels was used, so that all 32 RF channels were tested.

### 4.5. Single Channel RF Heating 

**Figure 9:**
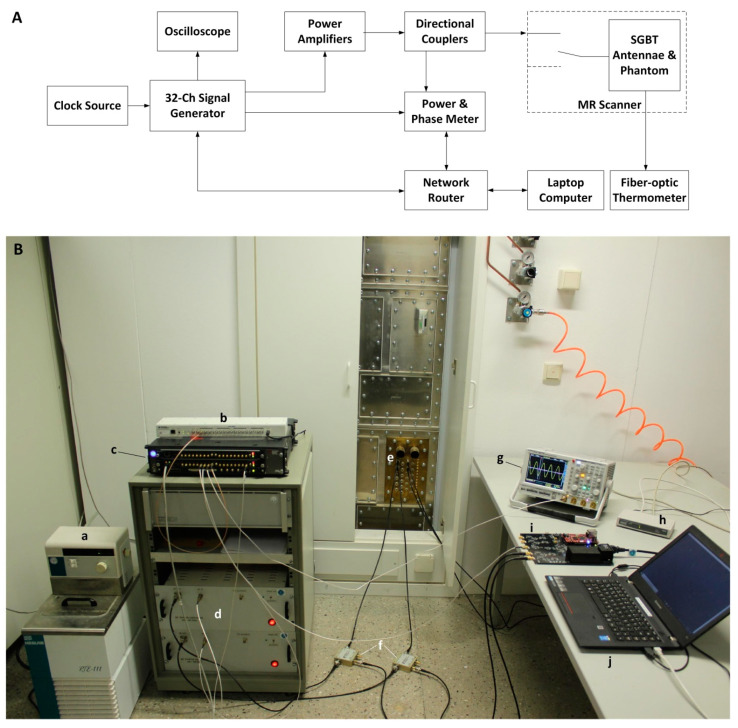
(**A**) Schematic of the experimental setup. Four RF signals were generated. One signal was connected to an oscilloscope for monitoring. Two signals were connected to RFPAs to drive the SGBT antennae and the last one was connected to the home-built power and phase meter as a reference signal. The forward coupled outputs of the directional couplers were fed to the power and phase meter. The signal generator, the power and phase meter and the laptop computer communicate with each other through network connections. (**B**) Setup used for the RF heating experiments comprising: (**a**) water cooling system, (**b**) clock distributor, (**c**) RF signal generator, (**d**) home-built RF power amplifiers, (**e**) penetration panel, (**f**) directional couplers, (**g**) oscilloscope, (**h**) network router, (**i**) home-built power and phase meter, (**j**) laptop for interacting with the equipment. The MR scanner and the fiber optic thermometer are not shown in the photo.

**Figure 10:**
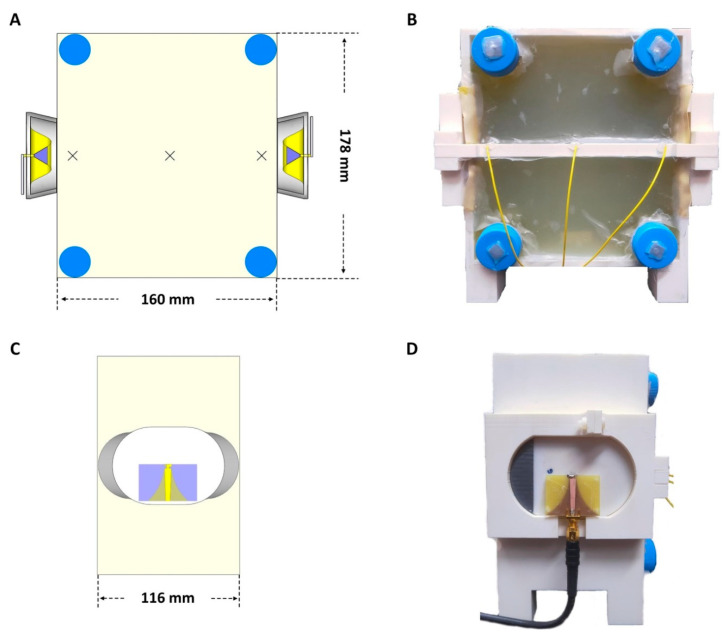
(**A**) The schematic of the front view (a transversal slice in the middle of the phantom) of the rectangular agarose phantom. The cross marks indicate the positions of fiber optic temperature sensors. (**B**) Front view of the rectangular agarose phantom: the yellow wires are fiber optic temperature sensors which were placed into the center of the phantom; the four blue tubes were filled with vegetable oil used as references for MR thermometry; the two antennae were installed opposing each other at two sides of the phantom. (**C**) The schematic of the lateral view of the phantom. (**D**) Lateral view of the phantom that shows one of the SGBT antennae. The printed circuit board on the antenna block is a balanced to unbalanced (balun) transformer for matching a 50 Ohm coaxial cable to the antenna port and vice-versa. A microstrip exponential taper was used to combine the balun with impedance matching.

Single channel heating experiments with the signal generator were conducted at 300 MHz, 400 MHz and 500 MHz. The output RF signal from channel one was fed to a home-built RF power amplifier (RFPA). The amplified signal was connected to an SGBT antenna through a directional coupler (BDC0810-50/1500, BONN Elektronik, Holzkirchen, Germany) and the feed through penetration panel of the MR scanner room. By adjusting the amplitude settings of channel one, a 42.5 dBm (17.78 W) signal was generated at the feeding port of the SGBT antenna. The power level was monitored by checking the forward coupled signal output of the directional coupler with a home-built power and phase meter. Figure 9 demonstrates the experimental setup. The antenna was applied to a muscle-mimicking agarose phantom (Figure 10, length = 160 mm, width = 116 mm, height = 178 mm, density = 1231.77 g/L, heat capacity = 3.00 (J/g)/K, thermal conductivity = 0.43 W/(m*K), NaCl: 5.48 g, Sugar: 2601 g, Agar: 52 g, Deionized H_2_O: 2600 g, CuSo_4_: 1.95 g [61]) placed in the isocenter of a 7.0 T human MR scanner (Magnetom, Siemens Healthineers, Erlangen, Germany). Table 3 summarizes the dielectric properties of this phantom, which were derived from the S-matrix measurement data using a vector network analyzer (ZVT 8, R&S, Munich, Germany). For benchmarking experimental data with numerical simulations, temperature simulations were performed in CST Microwave Studio 2018. For this purpose, the phantom configuration used in the heating experiments was incorporated into the numerical simulations. The CST thermal transient solver was adopted with a hexahedral mesh type. The maximum mesh size was set to 2.0 × 2.0 × 2.0 mm³, which is sufficient for the problem that decreasing the maximum mesh size by 10% does not yield substantial changes (<0.01%) in the simulation results. We used open boundary condition with the ambient temperature 20 °C set at the boundary as constant temperature. The simulation started with an initial temperature of 20 °C. RF heating with P_in_ = 17.78 W at the feeding port of the SGBT antenna and a duration of 10 minutes was applied for 300 MHz, 400 MHz and 500 MHz. MR thermometry using the PRFS approach [29,62,63] (TR = 99 ms, TE1 = 2.73 ms, TE2 = 6.71 ms, voxel size = 1 × 1 × 5 mm³) at 297.2 MHz was conducted before and after the RF heating for each frequency. Vegetable oil was used as a reference to correct the magnetic field drifts [64]. Fiber optic temperature sensors (Neoptix, Quebec, QC, Canada) were used to validate the MRT results. The heating experiments were repeated with the signal generator replaced by a commercial one (SMGL, R&S) to compare the results.

### 4.6. Dual Channel RF Heating 

The RF heating experiment was extended to two channels to demonstrate the synthesizer’s ability to accurately adjust the phase of the RF signals. Channel two was added to the setup of the single channel heating experiment. Both channels were set to generate 400 MHz RF signals. Each channel was connected to a home-built RF power amplifier. The outputs from the RFPAs were fed to two SGBT antennae through directional couplers and the feed through penetration panel. By adjusting the amplitude settings of the synthesizer, the RF power level at the feeding ports of the SGBT antennae was set to 42.5 dBm (17.78 W) for each channel. The antennae were positioned opposite to each other and applied to the same phantom used for the single channel experiments (Figure 10). The phantom was placed in the isocenter of the 7.0 T MR scanner. The experimental setup is illustrated in Figure 9. The two forward coupled outputs from the directional couplers were connected to a home-built power and phase meter to monitor the power level and phase relationship of the two channels. Three heating experiments were conducted using three phase shifts (ϕ = 0°, ϕ = 90° and ϕ = 180°) added to channel one. RF heating (P_in_ = 17.78 W at the feeding port of the SGBT antenna, t = 10 min) was applied for each phase setting. For benchmarking experimental data with numerical simulations, temperature simulations with the same frequency, power and phase settings were performed in CST Microwave Studio 2018. The CST thermal transient solver was adopted with a hexahedral mesh type. The maximum mesh size was set to 2.0 × 2.0 × 2.0 mm³, which is sufficient for the problem that decreasing the maximum mesh size by 10% does not yield substantial changes (<0.01%) in the simulation results. We used open boundary condition with the ambient temperature 20 °C set at the boundary as constant temperature. The simulation started with an initial temperature of 20 °C. MRT using the PRFS approach (TR = 99 ms, TE1 = 2.73 ms, TE2 = 6.71 ms, voxel size = 1 × 1 × 5 mm³) at 297.2 MHz was conducted before and after the RF heating. Vegetable oil was used as a reference to correct the magnetic field drift. Fiber optic temperature sensors were used to validate the MRT results. The RF heating experiments were repeated with the signal generator replaced by a commercial high-end 4-channel arbitrary waveform generator (M8190A, Keysight) for comparison.

## 5. Conclusions

This work demonstrates the development, implementation, evaluation, validation and application of a 32-channel RF signal generator system tailored for RF-induced heating. The RF heating experiments demonstrated the efficacy of the RF signal generator, which is competitive with high-end commercial signal generators equipped with a lower number of RF channels. The large number of coherent RF channels, wide frequency range, accurate phase shift, and highly flexible configurations provided by the signal generator form a technological basis for future hyperthermia applications driven by ThermalMR.

## Figures and Tables

**Table 1:** Test results of the switching/settling times for frequency, amplitude and phase.

	Mean	Minimum	Maximum	Standard Deviation
**Frequency switching time (ms)**	2.208	1.872	2.582	0.219
**Amplitude settling time (μs)**	617	320	810	143
**Phase settling time (μs)**	196	140	290	40

**Table 2:** Test results of the phase shift experiments.

Phase Shift c (°)	Phase Reading a (°) before Shift	Phase Reading b (°) after Shift	Measured Shift b−a (°)	Phase Shift Error (c−(b−a)) mod 360 (°)
300 MHz				
0.5	0.5588	1.0553	0.4965	0.0035
1	0.9936	1.9973	1.0037	−0.0037
5	0.4629	5.459	4.9961	0.0039
10	1.0537	11.0544	10.0007	−0.0007
15	0.6694	15.7225	15.0531	−0.0531
45	0.6919	45.75	45.0581	−0.0581
90	0.2352	90.2764	90.0412	−0.0412
100	0.4318	100.4639	100.0321	−0.0321
180	0.4238	179.5796	180.0034	−0.0034
200	0.4315	−159.5768	−160.0083	0.0083
270	0.6984	−89.2514	−89.9498	−0.0502
300	0.4575	−59.5542	−60.0117	0.0117
600 MHz				
0.5	1.0714	1.5928	0.5214	−0.0214
1	1.0517	2.1267	1.075	−0.075
5	1.1045	6.1592	5.0547	−0.0547
10	1.0741	11.2004	10.1263	−0.1263
15	1.1124	16.1507	15.0383	−0.0383
45	1.19	46.254	45.064	−0.064
90	1.1915	91.185	89.9935	0.0065
100	1.1294	101.1894	100.06	−0.06
180	1.1242	−178.8824	−180.0066	0.0066
200	0.9942	−158.9036	−159.8978	−0.1022
270	1.082	−88.783	−89.865	−0.135
300	−1.8299	−61.7925	−59.9626	−0.0374
900 MHz				
0.5	−18.8026	−18.1841	0.6185	−0.1185
1	−18.8053	−17.6564	1.1489	−0.1489
5	−18.8533	−13.7494	5.1039	−0.1039
10	−18.9628	−8.8075	10.1553	−0.1553
15	−18.8442	−3.8403	15.0039	−0.0039
45	−19.2507	25.888	45.1387	−0.1387
90	−19.246	70.7953	90.0413	−0.0413
100	−19.2349	80.806	100.0409	−0.0409
180	−19.2853	160.8254	180.1107	−0.1107
200	−19.2892	−179.1758	−159.8866	−0.1134
270	−19.3033	−109.251	−89.9477	−0.0523
300	−17.7194	−77.6205	−59.9011	−0.0989

**Table 3:** Dielectric properties of the phantom at frequencies of 300 MHz, 400 MHz and 500 MHz.

	300 MHz	400 MHz	500 MHz
**Relative permittivity, ε_r_**	56.2091	54.3220	49.4599
**Conductivity, σ (S/m)**	0.1834	0.2535	0.3651

## Supplementary Materials

The following are available online at https://www.mdpi.com/2072-6694/12/7/1720/s1, Video S1: Live adjustments of the frequency of the RF signals. Video S2: The continuous adjustments of the signal’s amplitude. Video S3: Live adjustments of the phase of the RF signals. Video S4: Frequency switching time measurements. Video S5: Amplitude settling time measurements. Video S6: Phase settling time measurements.
